# Molecular Phenotyping and Genomic Characterization of a Novel Neuroactive Bacterium Strain, *Lactobacillus murinus* HU-1

**DOI:** 10.3389/fphar.2019.01162

**Published:** 2019-10-04

**Authors:** Yeonwoo Lebovitz, Michelle H. Theus

**Affiliations:** ^1^Graduate Program in Translational Biology, Medicine, and Health, Virginia Tech, Blacksburg, VA, United States; ^2^Department of Biomedical Sciences and Pathobiology, VA-MD College of Veterinary Medicine, Blacksburg, VA, United States; ^3^School of Neuroscience, Virginia Tech, Blacksburg, VA, United States; ^4^Center for Regenerative Medicine, VA-MD College of Veterinary Medicine, Blacksburg, VA, United States

**Keywords:** genome sequence, gut-brain axis, *Lactobacillus murinus*, microbiome, probiotic

## Introduction

Over a century ago, Elie Metchnikoff observed unusual longevity among Bulgarian populations that consumed soured milk containing lactic acid bacteria (Metchnikoff and Mitchell, 1908). He theorized that the production of lactic acid by such bacteria prevented “intestinal putrefaction” and popularized the deliberate consumption of *Lactobacilli*-cultured milk for health purposes (Cavaillon and Legout, 2016). Since Metchnikoff’s time, the contributions of *Lactobacilli* to host health have been greatly expanded to include roles in immune homeostasis, production of key nutrients and vitamins, and even as a physical barrier against infection by pathogenic microorganisms (Macfarlane and Macfarlane, 2012). Recent investigations into the gut–brain axis revealed possible additional functions of lactic acid bacteria in regulating mood and cognition when ingested orally as a probiotic supplement. Indeed, many *Lactobacillus* sp. have been correlated with improved psychological outcomes, especially for neurodevelopmental, mood, stress, and anxiety disorders (Bravo et al., 2011; Buffington et al., 2016; Liu et al., 2019; Marotta et al., 2019; Sgritta et al., 2019).

While the neurological mechanisms behind probiotic consumption have yet to be fully understood, current evidence suggests *Lactobacilli* likely confer mental health benefits through both direct and indirect pathways, such as vagal nerve signaling and T_reg_ regulation (Bravo et al., 2011; Wells, 2011). Gut bacterial production of known neurotransmitters, such as gamma-aminobutyric acid (GABA), serotonin, and glutamate (Lyte, 2011; Dienel, 2012; Steenbergen et al., 2015), as well as a newfound appreciation for neuroactive potential of common bacterial metabolites, such as lactate and short-chain fatty acids, further suggest additional pathways in which *Lactobacilli* may contribute to neurological health (Proia et al., 2016; Oleskin et al., 2017).

Previously, we reported neuroprotective effects of *Lactobacillus murinus* HU-1, a mutant strain isolated from mouse, in preventing development of premature senescence in cortical microglia and social behavior deficits in murine offspring reared under antibiotics-driven maternal microbiome dysbiosis (Lebovitz et al., 2019). A key component of a complete and diverse gut microbiome, *L. murinus* represents a commensal gut bacterium naturally found in the gut of healthy mammals, including rodents, dogs, pigs, and poultry (Kurzak et al., 1998; Greetham et al., 2002; Gardiner et al., 2004). Compared to other *Lactobacilli, L. murinus* is a relatively understudied species that only recently gained attention as a probiotic candidate, including potential applications regarding neonatal necrotizing enterocolitis (Isani et al., 2018), antimicrobial production (Nardi et al., 2005), pathogen antagonism (Vasconcelos et al., 2003), intestinal barrier function (Delucchi et al., 2017), food allergy (Huang et al., 2016), type 1 diabetes (Sane et al., 2018), hypertension (Wilck et al., 2017), age-associated inflammation (Pan et al., 2018), and bacterial translocation (Ma et al., 1990). Here, we characterize the genome of a novel strain, *L. murinus* HU-1, and profile its molecular features in an effort to better understand its influence on host physiology and neurobehavior.

## Materials and Methods

### Bacterial Isolation and Growth

*Lactobacillus murinus* HU-1 originally isolated from murine gut was maintained as frozen stock in 20% glycerol at −80 °C until needed. Frozen stock was directly cultured overnight in MRS broth or streaked onto MRS agar (Becton, Dickinson and Company, Franklin Lakes, NJ, USA) at 37 °C, as described (Kragh et al., 2018).

### Animals

All mice were housed in an AAALAC accredited, virus/specific antigen-free facility with a 12 h light-dark cycle; food (Teklad 2918, Envigo, Huntingdon, UK) and water provided *ad libitum*. Outbred CD-1 IGS mice were purchased from Charles River (Strain code 022, Charles River Laboratories, Wilmington, MA, USA), and inbred B6.129P-*Cx3cr1* *^tm1Litt^*/J mice (Stock no. 005582) were purchased from Jackson Laboratory (Jackson Laboratory, Bar Harbor, ME, USA). Experimental CD-1 mice were administered a single oral dose of *L. murinus* HU-1 (10^9^ CFU) and then maintained on an antibiotic cocktail of 0.4 mg/ml kanamycin, 850 U/ml colistin, 0.215 mg/ml metronidazole (Bio-World, Dublin, OH, USA), 0.035 mg/ml gentamicin (Vet One, Boise, ID, USA), and 0.045 mg/ml vancomycin (Hospira Inc., Lake Forest, 372 IL, USA) (ABX^HU-1^) or the above antibiotic cocktail with an addition of 0.5 mg/ml amoxicillin/clavulanic acid (Zoetis, Parsippany, NJ, USA) (ABX^HU-1+AC^). Antibiotics were administered *via* drinking water. All experiments were conducted in accordance with the NIH Guide for the Care and Use of Laboratory Animals and conducted under the approval of the Virginia Tech Institutional Animal Care and Use Committee (IACUC; #17-043).

### Murine Fecal Bacteria Identification and Antibiotic Susceptibility

Fresh fecal pellets from mice were collected into sterile 1.5 ml microcentrifuge tubes and submitted to the Virginia-Maryland College of Veterinary Medicine’s Animal Laboratory Services for identification of culturable bacteria and to undergo antibiotics susceptibility testing. In brief, murine fecal pellets were immediately cultured on MacConkey and chocolate agar overnight. Colony formations were scored and identified using Bruker Microflex Biotyper 3.1 MALDI-TOF (Bruker Daltonics, Billerica, MA, USA). Additional colonies were collected from pure cultures of identified bacteria and subjected to antibiotics susceptibility testing using Sensititre™ Complete Automated AST System (Thermo Fisher Scientific Solutions LLC, Waltham, MA, USA) according to manufacturer’s instructions.

### DNA Isolation and Whole Genome Sequencing

Genomic DNA was extracted and purified from *L. murinus* HU-1 isolates *via* kit (SKU D6010, Zymo Research, Irvine, CA, USA) and submitted to the Beijing Genomics Institute (Shenzhen, China) for whole genome re-sequencing. In brief, the genome was sequenced using an Illumina HiSeq 4000 system (Illumina, San Diego, CA, USA). Genomic DNA was sheared randomly to construct three read libraries with lengths of 300 bp by a Bioruptor ultrasonicator (Diagenode, Denville, NJ, USA) and physiochemical methods. The paired-end fragment libraries were sequenced according to manufacturer’s protocol. Raw reads of low quality from paired-end sequencing were discarded.

### Genome Assembly, Annotation, and Genomic Features

Bioinformatic analyses on *L. murinus* HU-1 were performed using Pathosystems Resource Integration Center (PATRIC) Comprehensive Genome Analysis service (Wattam et al., 2017). In brief, raw sequenced reads were assembled using SPAdes. Assembled genome was then annotated using RAST tool kit (RASTtk). Specialty genes were determined by homology to those identified as drug targets in the DrugBank database (Law et al., 2014), transporters in the Transporters Classification Database (TCDB) (Saier et al., 2016), and virulence factors in the Virulence Factor Database (VFDB) (Chen et al., 2016). Antibiotic resistance genes, their functional annotation, mechanism of antibiotic resistance, and drug class were identified using the Comprehensive Antibiotic Resistance Database (McArthur et al., 2013) and a curated database of representative antibiotic resistance gene sequence variants available on PATRIC (Wattam et al., 2017). Subsystems analysis depicting biological processes or structural complexes of specific genes was based on SEED subsystems annotations (Overbeek et al., 2005). A comprehensive genome analysis was similarly performed for the representative strain, *L. murinus* ASF361 (SRR769344), to provide a basis for comparison.

### Phylogenetic Tree of *L. murinus* Strains

Phylogenetic tree of *L. murinus* HU-1 and 10 publicly available *L. murinus* whole genome sequences [strains: ASF361 (representative strain), 510-9, CR141, CR147, DSM 20452 = NBRC 14221, EF-1, KM-1, UBA3408, UBA3411, UBA7190] was constructed using PATRIC codon tree method utilizing PATRIC PGFams as homology groups and analyzing aligned proteins and coding DNA from single-copy genes using the program RAxML version 8.2.11 and fast bootstrapping to provide support values in the tree (Davis et al., 2016).

### Proteomic Analysis

Assessment of protein-coding genes in *L. murinus* HU-1 was constructed using the Protein Family Sorter Service (PATtyFams) tool in PATRIC. In brief, protein families were generated based on k-mer functional assignments using RAST and Markov Cluster algorithm (MCL) (Davis et al., 2016). PATRIC genus-specific families (PLfams) option was used to provide comparative assessment of protein families between *L. murinus* HU-1 and relevant strains due to the stringent criteria used (MCL inflation = 3.0), which allow for greater specificity when comparing genomes within the same species.

## Results

### Genomic Features of *L. murinus* HU-1

**Figure 1:**
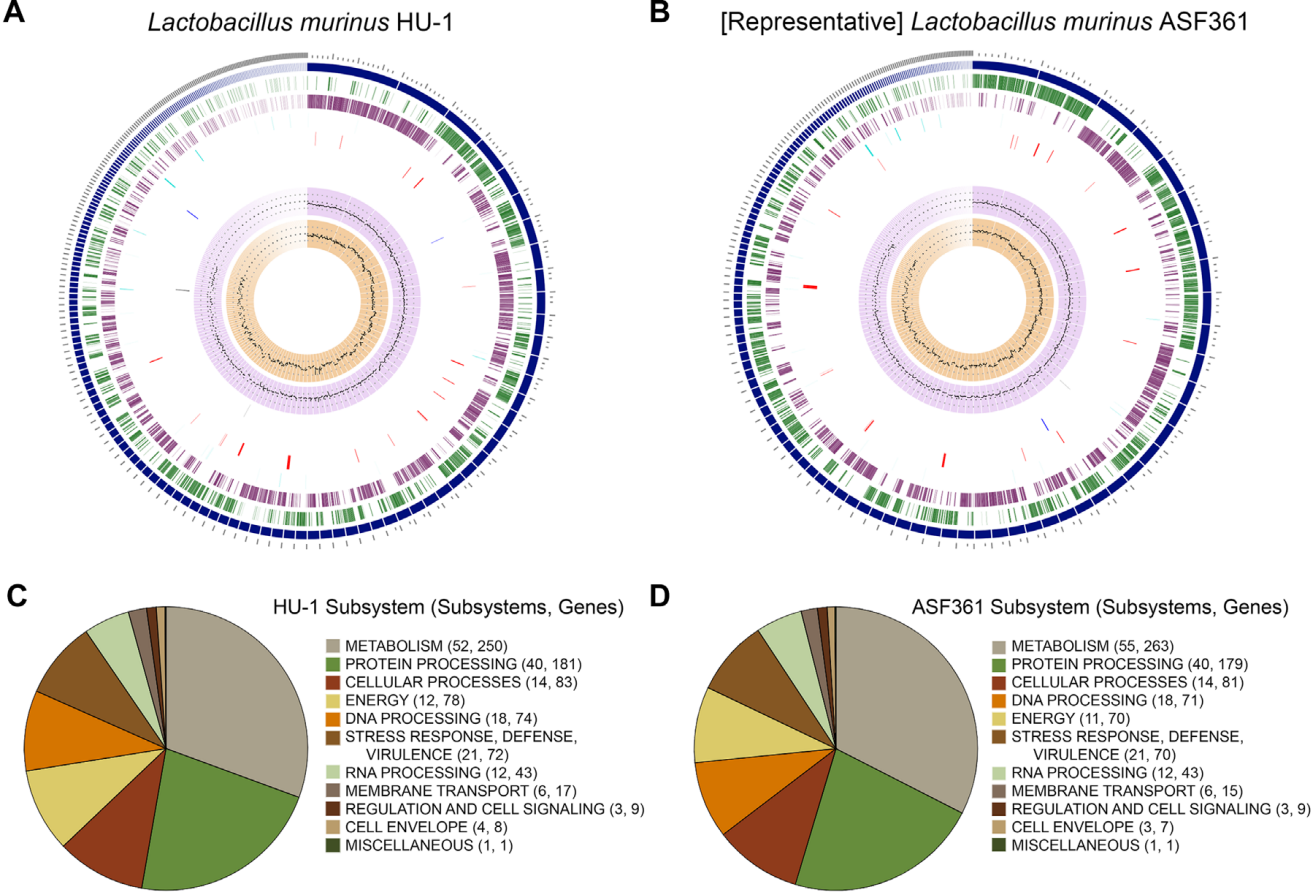
Comparative genomic characterization of novel strain, *Lactobacillus murinus* HU-1. **(A–B)** Circular graphic of novel strain, *L. murinus* HU-1, **(A)** and representative strain, *L. murinus* ASF361 **(B)**. From outer to inner rings are: contigs, coding sequence (CDS) on the forward strand, CDS on the reverse strand, RNA genes, CDS with homology to known antimicrobial resistance genes, CDS with homology to known transporters, CDS with homology to known drug targets, GC content, and GC skew. **(C–D)** Pie charts indicating major subsystems and number of genes in each category for *L. murinus* HU-1 **(C)** and *L. murinus* ASF361 **(D)**. *L. murinus* HU-1 contained additional energy- and cell envelope-related genes specific to central metabolism and cell wall synthesis, while the representative strain contained additional metabolism-related genes specific to biotin synthesis, NAD and NADP cofactor biosynthesis, and thiamin transport.

To ascertain whether *Lactobacillus murinus* HU-1 was a novel strain, we conducted whole genome sequencing and performed comprehensive genome analysis using PATRIC (Wattam et al., 2017). Genome assembly analysis estimated genome length to be 2,408,429 bp, average GC content of 39.84%, and 232 contigs. Taxonomy was confirmed as *L. murinus*. Annotated genome analysis revealed 2,597 protein coding sequences (CDS), 55 transfer RNA (tRNA) genes, and 4 ribosomal RNA (rRNA) genes. Of these, 875 represented hypothetical proteins and 1,722 proteins with functional assignments, including 535 with Enzyme Commission (EC) numbers, 441 with Gene Ontology (GO) assignments, and 355 mapped to KEGG pathways. Investigation of specialty genes resulted in 2 potential drug targets (Law et al., 2014), 2 transporter genes (Saier et al., 2016), 23 potential antibiotic resistance genes (McArthur et al., 2013; Wattam et al., 2017), and no known virulence factors (Chen et al., 2016). These genomic features are visualized in a circular graphic in **Figure 1A**.

### Comparison Against Representative Strain, *L. murinus* ASF361

Next, we conducted comparative genome analysis against the representative genome, *L. murinus* ASF361 (SRR769346) (“representative strain”). We determined *L. murinus* ASF361 to be the representative strain based on its inclusion as one of the eight microbes making up the Altered Schaedler Flora, a defined collection of gut bacteria deemed to be necessary for maintaining murine health, and thereby its endemic nature in most laboratory mice (Wymore Brand et al., 2015). Investigation of specialty genes in the representative strain revealed 1 potential drug target, 1 transporter gene, 22 potential antibiotic resistance genes, and no known virulence factors. The representative strain genomic features are visualized in **Figure 1B**.

The specialty genes expressed in the representative genome were also shared by *L. murinus* HU-1. Specifically, these included *ptsH*, which encodes a potential drug target, phosphocarrier protein, Hpr (Jia et al., 1993), and a copper transporter, *tcrB* (Hasman, 2005). Potential antibiotic resistance genes were broadly determined by PATRIC as any sequence variant whose presence/absence/mutation were related to antibiotic resistance and categorized according to the following mechanisms: antibiotic target in susceptible species (*alr*, *ddl*, *EF-G*, *EF-Tu*, *folA*, *dfr*, *gyrA*, *gyrB*, *inhA*, *fabI*, *iso-tRNA*, *kasA*, *murA*, *rho*, *rpoB*, *rpoC*, *s10p*, *s12p*); antibiotic target modifying enzyme (*rlmA[II]*); gene conferring resistance *via* absence (*gidB*); and protein altering cell wall charge conferring antibiotic resistance (*mprF*, *pgsA*) (Wattam et al., 2017). Notably, assessment of antibiotic resistance genes according to Comprehensive Antibiotic Resistance Database (CARD) identified only *EF-Tu* as a potential antibiotic resistance gene (McArthur et al., 2013). In addition to the specialty genes identified in the representative genome, *L. murinus* HU-1 differentially possessed a multiple sugar ABC transporter gene, *msmG* (Webb et al., 2008), a potential drug target related to galactose metabolism, *lacG* (Wiesmann et al., 1997), and an extra copy of the potential antibiotic resistance gene, *inhA*/*fabI* (Lu and Tonge, 2008).

### Comparative Characterization of Subsystems Categories

Subsystems analysis of *L. murinus* HU-1 and the representative genome showed similar categorization of biological processes and pathways, including the majority of gene functions allocated to metabolism and protein processing (**Figures 1C, D**). *L. murinus* HU-1 genes included additional energy-related genes specific to dihydroxyacetone kinase (DhaK) with purported functional involvement in central metabolism (Erni et al., 2006), as well as a cell wall-related gene specific to dTDP-rhamnose synthesis (van der Beek et al., 2019). In contrast, the representative genome differentially included additional metabolism-related genes specific to biotin synthesis and utilization (Satiaputra et al., 2016), NAD and NADP cofactor biosynthesis (Gazzaniga et al., 2009), and thiamin transport (Rodionov et al., 2002).

### Proteomic Assessment of *L. murinus* HU-1

**Figure 2:**
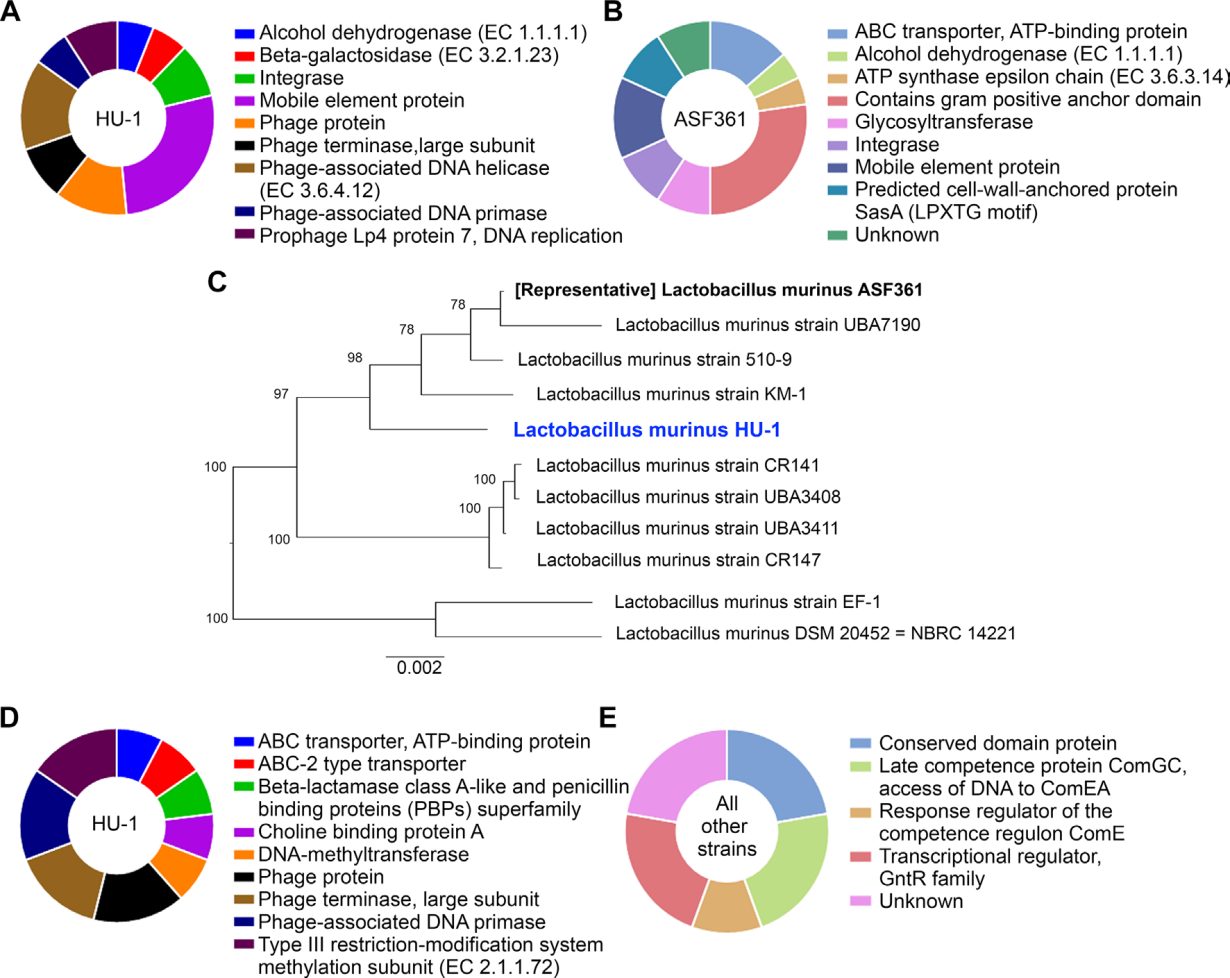
*L. murinus* HU-1 maintains features distinct from other strains of *L. murinus*. **(A)** Attributed protein families identified in *L. murinus* HU-1 only that were not in the representative strain, *L. murinus* ASF361. Pie chart excludes hypothetical proteins, which were in the majority. **(B)** Attributed protein families identified in the representative strain only that were not in *L. murinus* HU-1, excluding hypothetical proteins. **(C)** Phylogenetic tree of *L. murinus* HU-1 compared to 10 other publicly available *L. murinus* strains on NCBI database. **(D)** Attributed protein families identified in *L. murinus* HU-1 that were not in the 10 other strains, excluding hypothetical proteins. **(E)** Only hypothetical proteins were found to be missing from *L. murinus* HU-1 compared to 10 other strains, but some of these proteins possessed hypothetical functional attributions.

To elucidate potential functional differences found in *L. murinus* HU-1 in comparison to the representative strain, we conducted comparative examination of the distribution of protein families in the two respective genomes *via* PATRIC genus-specific families (PLfams) (Davis et al., 2016). We observed the presence of 378 protein families in *L. murinus* HU-1 that were not identified in the representative genome; 259 of these were for hypothetical proteins. Of the attributed protein families only, approximately 51.5% were functionally related to phage-specific activities and the remaining protein families were distributed across mobile element protein, integrase, alcohol dehydrogenase, and beta-galactosidase activity (**Figure 2A**). The latter is a critical enzyme produced by infant gut bacteria and is a common feature of probiotic *Bifidobacteria* (Milani et al., 2017). Prophage proteins identified in this genome (Lp2 protein 4, Lp4 protein 7, ps1 protein 14, and ps3 protein 13) were previously found in other probiotic strains, *L. reuteri*, *L. plantarum*, and *Lactococcus lactis* (UniProt, 2019). Lp2 and Lp4 were considered non-inducible prophages, whereas ps1 and ps3 were predicted to be related to DNA packaging (Bolotin et al., 2001; Ventura et al., 2003). In contrast, we observed 163 protein families in the representative genome that were not identified in *L. murinus* HU-1, including 125 hypothetical proteins. Of the attributed protein families found in the representative strain only, approximately 30% were functionally related to gram positive anchor domain and the rest distributed across assorted activities (**Figure 2B**).

### Comparison Against Other Sequenced *L. murinus* Strains

Previously, we and others described *L. murinus* as phylogenetically closest to other probiotic strains, *L. animalis* and *L. salivarius* (Pan et al., 2018; Lebovitz et al., 2019). To contextualize *L. murinus* HU-1 within its subspecies, we constructed a phylogenetic tree using whole genomes of 10 publicly available *L. murinus* strains, including the representative strain (**Figure 2C**). The tree formed three main branches with *L. murinus* HU-1 clustering with the representative strain, albeit several nodes apart. Protein families analysis comparing *L. murinus* HU-1 to these other 10 genomes revealed that *L. murinus* HU-1 possessed 55 protein families not found in the other strains. The majority of these genes belonged to unattributed hypothetical proteins, although the second largest proportion of genes encoded for proteins relevant to phages (approximately 46%) while the rest belonged to ABC transporters, beta-lactamase binding protein, choline binding protein, and methyltransferases (**Figure 2D**). In contrast, *L. murinus* HU-1 was shown to be just missing 7 hypothetical protein families otherwise found in the 10 other strains. These were mostly of unattributed or unknown function, but several were purported to be related to a conserved domain protein or regulatory competence proteins (**Figure 2E**).

### Assessment of Antibiotic Susceptibility of *L. murinus* HU-1

In support of the potential antibiotic resistance genes identified in the above genomic analysis, we isolated *L. murinus* from antibiotics-treated experimental and conventionally-raised CD-1 mice feces for antibiotic sensitivity testing *via* disk diffusion method. Only *L. murinus* could be isolated from experimental mice harboring *L. murinus* HU-1 (ABX^HU-1^) and these isolates exhibited antibiotic resistance to amikacin and gentamicin. Introducing amoxicillin/clavulanic acid to the antibiotic cocktail for two weeks (ABX^HU-1+AC^) still resulted in *L. murinus* growth, however, it no longer exhibited antibiotic resistance according to the disk diffusion assay (**Supplemental Table 1**). Meanwhile, native *L. murinus* isolates from conventionally-raised control mice did not exhibit antibiotic resistance. Interestingly, native *L. murinus* isolates from conventionally-raised B6.Cx3cr1 mice, which were a different strain and purchased from a different vendor than the CD-1 mice, exhibited widespread resistance to amikacin, cefazolin, chloramphenicol, clindamycin, erythromycin, gentamicin, and imipenem. Furthermore, cross-rearing conventional B6.Cx3cr1 offspring with conventional CD-1 mice resulted in *L. murinus* isolates that no longer maintained antibiotic resistance traits (**Supplemental Table 1**). Thus, *L. murinus* HU-1 isolated from experimental mouse feces exhibited antibiotic resistance as predicted in the genomic analysis, but this trait was malleable under additional antibiotic therapy and was not unique as native *L. murinus* found in conventionally-housed mice also exhibited antibiotic resistance from the outset.

## Conclusion

*Lactobacillus murinus* represents a promising probiotic candidate with a wide range of potential health applications. Here, we sequenced and analyzed the whole genome of a novel strain, *L. murinus* HU-1, previously reported to confer neurodevelopmental benefits in a murine model of maternal microbiome dysbiosis. Notably, *L. murinus* HU-1 expressed genes specific to beta-galactosidase production, which may counteract the microglial accumulation of this enzyme typically found in neurological disease models of premature cellular senescence. Beta-galactosidase production is also a common trait of commensal bacteria found in the healthy infant gut, as it is a key enzyme for proper digestion of mammary milk. Compared to other publicly available *L. murinus* strains, *L. murinus* HU-1 shared important traits of probiotics, such as expression of genes related to bacteriocin activity and resistance to a variety of environmental stresses. However, *L. murinus* HU-1 uniquely expressed genes specific to prophage activity, potential antibiotic resistance, and select biological processes. The impact of phages in probiotic genomes remain a nascent area of study; some have been credited with enhanced fitness to the gastrointestinal niche while others are considered problematic for the fermentative dairy industry due to potential phage predation. As the phages identified in *L. murinus* HU-1 were not associated with virulence, it is possible that their presence may contribute to host health through yet unknown adaptive advantages. Additional study into *L. murinus* HU-1 interactions with the host, as well as detailed conditions for its growth and scalability, will be needed to demonstrate probiotic efficacy and safety in the future. 

## Data Availability Statement

This Whole Genome Shotgun project has been deposited at DDBJ/ENA/GenBank under the accession VMDX00000000. The version described in this paper is version VMDX01000000.

## Ethics Statement

The animal study was reviewed and approved by Virginia Tech IACUC.

## Author Contributions

YL and MT performed research and analyzed data. YL wrote paper. MT wrote and edited paper, designed research, and contributed reagents/analytic tools.

## Funding

This work was supported by the Center for One Health Research Seed Grant (#459719) at Virginia-Maryland College of Veterinary Medicine and The Edward Via College of Osteopathic Medicine (MT), Virginia Tech Center for Autism Research Student Award (YL), and Virginia Tech Graduate Research Development Program Award (YL).

## Conflict of Interest

The authors declare that the research was conducted in the absence of any commercial or financial relationships that could be construed as a potential conflict of interest.

## Supplementary Material

Supplemental Table 1 Comparative antibiotic susceptibility of *L. murinus* HU-1 versus native *L. murinus* isolated from mice feces. Disk diffusion antibiotic susceptibility assay results performed on *L. murinus* cultures isolated from feces of experimental mice (ABX^HU-1^, ABX^HU-1+AC^) and conventionally-raised controls (CONV). *L. murinus* isolated from ABX^HU-1^ mice showed antibiotic resistance to amikacin and gentamicin. Supplementing ABX^HU-1^ mice with amoxicillin/clavulanic acid (ABX^HU-1+AC^) for two weeks resulted in loss of antibiotic resistance in *L. murinus* isolates. Isolates collected from CONV mice showed variable antibiotic resistance based on mouse strain and vendor origin. Assay results are interpreted accordingly: S = Sensitive; I = Intermediate; R = Resistant; NI = Not Interpreted; N/A = Not available.

## References

[B1] Bolotin A. Wincker P. Mauger S. Jaillon O. Malarme K. Weissenbach J. (2001). The complete genome sequence of the lactic acid bacterium *Lactococcus lactis* ssp. lactis IL1403. Genome Res. 11 (5), 731–753. 10.1101/gr.GR-1697R 11337471 PMC311110

[B2] Bravo J. A. Forsythe P. Chew M. V. Escaravage E. Savignac H. M. Dinan T. G. (2011). Ingestion of *Lactobacillus* strain regulates emotional behavior and central GABA receptor expression in a mouse *via the* vagus nerve. Proc. Natl. Acad. Sci. U.S.A. 108 (38), 16050–16055. 10.1073/pnas.1102999108 21876150 PMC3179073

[B3] Buffington S. A. Di Prisco G. V. Auchtung T. A. Ajami N. J. Petrosino J. F. Costa-Mattioli M. (2016). Microbial reconstitution reverses maternal diet-induced social and synaptic deficits in offspring. Cell 165 (7), 1762–1775. 10.1016/j.cell.2016.06.001 27315483 PMC5102250

[B4] Cavaillon J. M. Legout S. (2016). Centenary of the death of Elie Metchnikoff: a visionary and an outstanding team leader. Microbes Infect. 18 (10), 577–594. 10.1016/j.micinf.2016.05.008 27288152

[B5] Chen L. Zheng D. Liu B. Yang J. Jin Q. (2016). VFDB 2016: hierarchical and refined dataset for big data analysis—10 years on. Nucleic Acids Res. 44 (D1), D694–D697. 10.1093/nar/gkv1239 PMC4702877 26578559

[B6] Davis J. J. Gerdes S. Olsen G. J. Olson R. Pusch G. D. Shukla M. (2016). PATtyFams: protein families for the microbial genomes in the PATRIC database. Front. Microbiol. 7, 118. 10.3389/fmicb.2016.00118 26903996 PMC4744870

[B7] Delucchi L. Fraga M. Zunino P. (2017). Effect of the probiotic *Lactobacillus murinus* LbP2 on clinical parameters of d1ogs with distemper-associated diarrhea. Can. J. Vet. Res. 81 (2), 118–121. 28408779 PMC5370537

[B8] Dienel G. A. (2012). Brain lactate metabolism: the discoveries and the controversies. J. Cereb. Blood Flow Metab. 32 (7), 1107–1138. 10.1038/jcbfm.2011.175 22186669 PMC3390802

[B9] Erni B. Siebold C. Christen S. Srinivas A. Oberholzer A. Baumann U. (2006). Small substrate, big surprise: fold, function and phylogeny of dihydroxyacetone kinases. Cell. Mol. Life. Sci. 63 (7–8), 890–900. 10.1007/s00018-005-5518-0 16505971 PMC11136353

[B10] Gardiner G. E. Casey P. G. Casey G. Lynch P. B. Lawlor P. G. Hill C. (2004). Relative ability of orally administered *Lactobacillus murinus* to predominate and persist in the porcine gastrointestinal tract. Appl. Environ. Microbiol. 70 (4), 1895–1906. 10.1128/AEM.70.4.1895-1906.2004 15066778 PMC383152

[B11] Gazzaniga F. Stebbins R. Chang S. Z. McPeek M. A. Brenner C. (2009). Microbial NAD metabolism: lessons from comparative genomics. Microbiol. Mol. Biol. Rev. 73 (3), 529–541. 10.1128/MMBR.00042-08 19721089 PMC2738131

[B12] Greetham H. L. Giffard C. Hutson R. A. Collins M. D. Gibson G. R. (2002). Bacteriology of the Labrador dog gut: a cultural and genotypic approach. J. Appl. Microbiol. 93 (4), 640–646. 10.1046/j.1365-2672.2002.01724.x 12234347

[B13] Hasman H. (2005). The tcrB gene is part of the tcrYAZB operon conferring copper resistance in *Enterococcus faecium* and *Enterococcus faecalis* . Microbiology 151 (Pt 9), 3019–3025. 10.1099/mic.0.28109-0 16151212

[B14] Huang C. H. Shen C. C. Liang Y. C. Jan T. R. (2016). The probiotic activity of *Lactobacillus murinus* against food allergy. J. Funct. Foods 25, 231–241. 10.1016/j.jff.2016.06.006

[B15] Isani M. Bell B. A. Delaplain P. T. Bowling J. D. Golden J. M. Elizee M. (2018). *Lactobacillus murinus* HF12 colonizes neonatal gut and protects rats from necrotizing enterocolitis. PLoS One 13 (6), e0196710. 10.1371/journal.pone.0196710 PMC6014650 29933378

[B16] Jia Z. Vandonselaar M. Quail J. W. Delbaere L. T. (1993). Active-centre torsion-angle strain revealed in 1.6 A-resolution structure of histidine-containing phosphocarrier protein. Nature 361 (6407), 94–97. 10.1038/361094a0 8421502

[B17] Kragh K. N. Alhede M. Rybtke M. Stavnsberg C. Jensen P. O. Tolker-Nielsen T. (2018). The inoculation method could impact the outcome of microbiological experiments. Appl. Environ. Microbiol. 84 (5), e02264–e02217. 10.1128/AEM.02264-17 PMC5812950 29269495

[B18] Kurzak P. Ehrmann M. A. Vogel R. F. (1998). Diversity of lactic acid bacteria associated with ducks. Syst. Appl. Microbiol. 21 (4), 588–592. 10.1016/S0723-2020(98)80071-4 9924827

[B19] Law V. Knox C. Djoumbou Y. Jewison T. Guo A. C. Liu Y. (2014). DrugBank 4.0: shedding new light on drug metabolism. Nucleic Acids Res. 42 (Database issue), D1091–D1097. 10.1093/nar/gkt1068 24203711 PMC3965102

[B20] Lebovitz Y. Kowalski E. A. Wang X. Kelly C. Lee M. McDonald V. (2019). Lactobacillus rescues postnatal neurobehavioral and microglial dysfunction in a model of maternal microbiome dysbiosis. Brain Behav Immun. 81, 617–629. 10.1016/j.bbi.2019.07.025 31351186

[B21] Liu R. T. Walsh R. F. L. Sheehan A. E. (2019). Prebiotics and probiotics for depression and anxiety: a systematic review and meta-analysis of controlled clinical trials. Neurosci. Biobehav. Rev. 102, 13–23. 10.1016/j.neubiorev.2019.03.023 31004628 PMC6584030

[B22] Lu H. Tonge P. J. (2008). Inhibitors of FabI, an enzyme drug target in the bacterial fatty acid biosynthesis pathway. Acc. Chem. Res. 41 (1), 11–20. 10.1021/ar700156e 18193820

[B23] Lyte M. (2011). Probiotics function mechanistically as delivery vehicles for neuroactive compounds: microbial endocrinology in the design and use of probiotics. Bioessays 33 (8), 574–581. 10.1002/bies.201100024 21732396

[B24] Ma L. Deitch E. Specian R. Steffen E. Berg R. (1990). Translocation of *Lactobacillus murinus* from the gastrointestinal tract. Curr. Microbiol. 20 (3), 177–184. 10.1007/BF02091994

[B25] Macfarlane G. T. Macfarlane S. (2012). Bacteria, colonic fermentation, and gastrointestinal health. J. AOAC Int. 95 (1), 50–60. 10.5740/jaoacint.SGE_Macfarlane 22468341

[B26] Marotta A. Sarno E. Del Casale A. Pane M. Mogna L. Amoruso A. (2019). Effects of probiotics on cognitive reactivity, mood, and sleep quality. Front. Psychiatry 10, 164. 10.3389/fpsyt.2019.00164 30971965 PMC6445894

[B27] McArthur A. G. Waglechner N. Nizam F. Yan A. Azad M. A. Baylay A. J. (2013). The comprehensive antibiotic resistance database. Antimicrob. Agents Chemother. 57 (7), 3348–3357. 10.1128/AAC.00419-13 23650175 PMC3697360

[B28] Metchnikoff E. Mitchell P. C. (1908). The prolongation of life; optimistic studies. New York & London: G.P. Putnam’s Sons.

[B29] Milani C. Duranti S. Bottacini F. Casey E. Turroni F. Mahony J. (2017). The first microbial colonizers of the human gut: composition, activities, and health implications of the infant gut microbiota. Microbiol. Mol. Biol. Rev. 81 (4), e00036–e00017. 10.1128/MMBR.00036-17 PMC5706746 29118049

[B30] Nardi R. M. Santoro M. M. Oliveira J. S. Pimenta A. M. Ferraz V. P. Benchetrit L. C. (2005). Purification and molecular characterization of antibacterial compounds produced by *Lactobacillus murinus* strain L1. J. Appl. Microbiol. 99 (3), 649–656. 10.1111/j.1365-2672.2005.02632.x 16108807

[B31] Oleskin A. V. Shenderov B. A. Rogovsky V. S. (2017). Role of neurochemicals in the interaction between the microbiota and the immune and the nervous system of the host organism. Probiotics Antimicrob. Proteins 9 (3), 215–234. 10.1007/s12602-017-9262-1 28229287

[B32] Overbeek R. Begley T. Butler R. M. Choudhuri J. V. Chuang H. Y. Cohoon M. (2005). The subsystems approach to genome annotation and its use in the project to annotate 1000 genomes. Nucleic Acids Res. 33 (17), 5691–5702. 10.1093/nar/gki866 16214803 PMC1251668

[B33] Pan F. Zhang L. Li M. Hu Y. Zeng B. Yuan H. (2018). Predominant gut *Lactobacillus murinus* strain mediates anti-inflammaging effects in calorie-restricted mice. Microbiome 6 (1), 54. 10.1186/s40168-018-0440-5 29562943 PMC5863386

[B34] Proia P. Di Liegro C. M. Schiera G. Fricano A. Di Liegro I. (2016). Lactate as a metabolite and a regulator in the central nervous system. Int. J. Mol. Sci. 17 (9), 1450. 10.3390/ijms17091450 PMC5037729 27598136

[B35] Rodionov D. A. Vitreschak A. G. Mironov A. A. Gelfand M. S. (2002). Comparative genomics of thiamin biosynthesis in procaryotes. New genes and regulatory mechanisms. J. Biol. Chem. 277 (50), 48949–48959. 10.1074/jbc.M208965200 12376536

[B36] Saier M. H. Jr. Reddy V. S. Tsu B. V. Ahmed M. S. Li C. Moreno-Hagelsieb G. (2016). The Transporter Classification Database (TCDB): recent advances. Nucleic Acids Res. 44 (D1), D372–D379. 10.1093/nar/gkv1103 26546518 PMC4702804

[B37] Sane F. Scuotto A. Pierrat V. Kacet N. Hober D. Romond M. B. (2018). Diabetes progression and alterations in gut bacterial translocation: prevention by diet supplementation with human milk in NOD mice. J. Nutr. Biochem. 62, 108–122. 10.1016/j.jnutbio.2018.08.017 30292969

[B38] Satiaputra J. Shearwin K. E. Booker G. W. Polyak S. W. (2016). Mechanisms of biotin-regulated gene expression in microbes. Synth. Syst. Biotechnol. 1 (1), 17–24. 10.1016/j.synbio.2016.01.005 29062923 PMC5640590

[B39] Sgritta M. Dooling S. W. Buffington S. A. Momin E. N. Francis M. B. Britton R. A. (2019). Mechanisms underlying microbial-mediated changes in social behavior in mouse models of autism spectrum disorder. Neuron 101(2) 246–259 e246. 10.1016/j.neuron.2018.11.018 30522820 PMC6645363

[B40] Steenbergen L. Sellaro R. van Hemert S. Bosch J. A. Colzato L. S. (2015). A randomized controlled trial to test the effect of multispecies probiotics on cognitive reactivity to sad mood. Brain Behav. Immun. 48, 258–264. 10.1016/j.bbi.2015.04.003 25862297

[B41] UniProt C. (2019). UniProt: a worldwide hub of protein knowledge. Nucleic Acids Res. 47 (D1), D506–D515. 10.1093/nar/gky1049 30395287 PMC6323992

[B42] van der Beek S. L. Zorzoli A. Canak E. Chapman R. N. Lucas K. Meyer B. H. (2019). Streptococcal dTDP-L-rhamnose biosynthesis enzymes: functional characterization and lead compound identification. Mol. Microbiol. 111 (4), 951–964. 10.1111/mmi.14197 30600561 PMC6487966

[B43] Vasconcelos A. L. S. Nicoli J. R. Nardi R. M. D. (2003). Antagonistic and protective effects against *Salmonella enterica* serovar Typhimurium by *Lactobacillus murinus* in the digestive tract of gnotobiotic mice. Braz. J. Microbio. 34, 21–24. 10.1590/S1517-83822003000500007

[B44] Ventura M. Canchaya C. Kleerebezem M. de Vos W. M. Siezen R. J. Brussow H. (2003). The prophage sequences of *Lactobacillus plantarum* strain WCFS1. Virology 316 (2), 245–255. 10.1016/j.virol.2003.08.019 14644607

[B45] Wattam A. R. Davis J. J. Assaf R. Boisvert S. Brettin T. Bun C. (2017). Improvements to PATRIC, the all-bacterial bioinformatics database and analysis resource center. Nucleic Acids Res. 45 (D1), D535–D542. 10.1093/nar/gkw1017 27899627 PMC5210524

[B46] Webb A. J. Homer K. A. Hosie A. H. (2008). Two closely related ABC transporters in *Streptococcus mutans* are involved in disaccharide and/or oligosaccharide uptake. J. Bacteriol. 190 (1), 168–178. 10.1128/JB.01509-07 17965163 PMC2223742

[B47] Wells J. M. (2011). Immunomodulatory mechanisms of lactobacilli. Microb. Cell Fact. 10 Suppl 1 1, S17. 10.1186/1475-2859-10-S1-S17 21995674 PMC3231924

[B48] Wiesmann C. Hengstenberg W. Schulz G. E. (1997). Crystal structures and mechanism of 6-phospho-beta-galactosidase from *Lactococcus lactis*. J. Mol. Biol. 269 (5), 851–860. 10.1006/jmbi.1997.1084 9223646

[B49] Wilck N. Matus M. G. Kearney S. M. Olesen S. W. Forslund K. Bartolomaeus H. (2017). Salt-responsive gut commensal modulates TH17 axis and disease. Nature 551 (7682), 585–589. 10.1038/nature24628 29143823 PMC6070150

[B50] Wymore Brand M. Wannemuehler M. J. Phillips G. J. Proctor A. Overstreet A. M. Jergens A. E. (2015). The altered Schaedler flora: continued applications of a defined murine microbial community. ILAR J. 56 (2), 169–178 10.1093/ilar/ilv012 26323627 PMC4554250

