# Supplementary material for: Molecular Phenotyping and Genomic Characterization of a Novel Neuroactive Bacterium Strain, Lactobacillus murinus HU-1
Source: Front Pharmacol. 2019 Oct 4;10:1162. doi: 10.3389/fphar.2019.01162 (PMC6787272; doi:10.3389/fphar.2019.01162)
Supplement: Supplemental Table 1 — Comparative antibiotic susceptibility of L. murinus HU-1 versus native L. murinus isolated from mice feces. Disk diffusion antibiotic susceptibility assay results performed on L. murinus cultures isolated from feces of experimental mice (ABXHU-1, ABXHU-1+AC) and conventionally-raised controls (CONV). L. murinus isolated from ABXHU-1 mice showed antibiotic resistance to amikacin and gentamicin. Supplementing ABXHU-1 mice with amoxicillin/clavulanic acid (ABXHU-1+AC) for two weeks resulted in loss of antibiotic resistance in L. murinus isolates. Isolates collected from CONV mice showed variable antibiotic resistance based on mouse strain and vendor origin. Assay results are interpreted accordingly: S = Sensitive; I = Intermediate; R = Resistant; NI = Not Interpreted; N/A = Not available. [file Table_1.docx]

| **Antibiotic** | **ABX^HU-1^ CD-1** | | **ABX^HU-1+AC^ CD-1** | | **CONV CD-1** | | **CONV B6.Cx3cr1** | | **CONV B6.Cx3cr1 offspring reared by CONV CD-1** | |
| --- | --- | --- | --- | --- | --- | --- | --- | --- | --- | --- |
| 1. Amikacin | >32.00 | R | <=4.00 | S | <=4.00 | S | >32.00 | R | <=4.00 | S |
| 1. Amoxicillin/ Clavulanic Acid | 0.25 | S | <=0.12 | S | 1 | S | >1.00 | NI | <=0.12 | S |
| 1. Ampicillin | <=0.12 | S | <=0.12 | S | N/A | N/A | N/A | N/A | 1 | S |
| 1. Cefazolin | <=1.00 | S | <=1.00 | S | 4 | I | 8 | R | <=1.00 | S |
| 1. Cefovecin | 2 | NI | <=0.25 | NI | >4.00 | NI | >4.00 | NI | <=0.25 | NI |
| 1. Cefoxitin | 8 | NI | <=2.00 | NI | 16 | NI | >16.00 | NI | <=2.00 | NI |
| 1. Cefpodoxime | <=2.00 | NI | <=2.00 | NI | >16.00 | NI | >16.00 | NI | <=2.00 | NI |
| 1. Ceftiofur | >4.00 | NI | <=0.25 | NI | <=0.25 | NI | >4.00 | NI | <=0.25 | NI |
| 1. Cephalothin | <=2.00 | S | <=2.00 | S | 4 | S | 8 | S | <=2.00 | S |
| 1. Chloramphenicol | <=4.00 | S | <=4.00 | S | 16 | I | >16.00 | R | <=4.00 | S |
| 1. Clindamycin | <=0.50 | S | <=0.50 | S | <=0.50 | S | >4.00 | R | <=0.50 | S |
| 1. Doxycycline | 4 | NI | <=2.00 | NI | 8 | NI | 8 | NI | <=2.00 | NI |
| 1. Enrofloxacin | 1 | NI | <=0.25 | NI | 1 | NI | >2.00 | NI | <=0.25 | NI |
| 1. Erythromycin | <=0.50 | S | <=0.50 | S | <=0.50 | S | >4.00 | R | <=0.50 | S |
| 1. Gentamicin | >8.00 | R | <=1.00 | S | <=1.00 | S | >8.00 | R | <=1.00 | S |
| 1. Imipenem | <=1.00 | S | <=1.00 | S | 2 | I | 8 | R | <=1.00 | S |
| 1. Marbofloxacin | 2 | NI | <=0.25 | NI | 2 | NI | >2.00 | NI | <=0.25 | NI |
| 1. Oxacillin + 2% NaCl | 1 | NI | <=0.25 | NI | >4.00 | NI | >4.00 | NI | <=0.25 | NI |
| 1. Penicillin | 0.5 | S | <=0.06 | S | >8.00 | NI | 8 | S | <=0.06 | S |
| 1. Rifampin | <=1.00 | NI | <=1.00 | NI | 2 | NI | >2.00 | NI | <=1.00 | NI |
| 1. Ticarcillin | <=8.00 | NI | <=8.00 | NI | <=8.00 | NI | >64.00 | NI | <=8.00 | NI |
| 1. Ticarcillin/ Clavulanic Acid | <=8.00 | NI | <=8.00 | NI | <=8.00 | NI | >64.00 | NI | <=8.00 | NI |
| 1. Trimethoprim/ Sulfamethoxazol | <=0.50 | NI | <=0.50 | NI | <=0.50 | NI | >2.00 | NI | <=0.50 | NI |
